# Clinical Outcome and Predictors of Intestinal Obstruction Surgery in Ethiopia: A Cross-Sectional Study

**DOI:** 10.1155/2020/7826519

**Published:** 2020-11-23

**Authors:** Tesfaye Derseh, Tariku Dingeta, Mohammed Yusouf, Binyam Minuye

**Affiliations:** ^1^Department of Obstetrics and Gynecology, College of Medicine and Health Science, Harar, Ethiopia; ^2^School of Public Health, College of Medicine and Health Sciences, Haramaya University, Harar, Ethiopia; ^3^College of Health Sciences, Debretabor University, Debretabor, Ethiopia

## Abstract

**Background:**

Despite the advancement in the healthcare system, the impact of surgical interventions on public health systems will continue to grow. But predicting the outcome is challenging. Concerns related to unexpected outcomes and delays in the diagnosis of postoperative complications are the major issue. Intestinal obstruction is a common life-threatening surgical condition followed by fatal and nonfatal postoperative complications. This study was aimed at assessing results after surgery for intestinal obstruction in a hospital of Ethiopia. *Methodology*. An institutional-based cross-sectional study was conducted among 254 postoperative patients admitted with intestinal obstruction from January 1, 2014, to December 31, 2017. Data were coded and entered into EpiData 4.2.0.0 software and exported to the Statistical Package for the Social Sciences version 22 for analysis. A binary logistic regression model was used for analysis. All variables with a *p* value < 0.25 during bivariable analysis were considered for multivariable logistic regression analysis.

**Results:**

The magnitude of poor surgical outcome of intestinal obstruction was 21.3% for patients enrolled into this investigation. The age group of ≥55 years (adjusted odds ratio (AOR) = 2.9, 95% CI: 1.03, 8.4), duration of illness of ≥24 hrs (AOR = 3.1, 95% CI: 1.03, 9.4), preoperative diagnosis of a gangrenous large bowel (AOR = 3.6, 95% CI: 1.3, 9.8), and a gangrenous small bowel (AOR = 4.2, 95% CI: 1.3, 13.7) were significantly associated with poor surgical outcome.

**Conclusions:**

The magnitude of poor surgical outcome was high. Age, late presentation of illness, and gangrenous bowel obstructions were significantly associated with poor outcomes. So, concern should be given in early detection and follow-up of patients who came late and older patients.

## 1. Background

Each year, millions of people undertake surgical interventions which account for an estimated 13% of the world's total disability-adjusted life years, 0.5-5% crude mortality rate, and 25% postoperative complications [1]. Intestinal obstruction is one of the surgical emergencies caused by a blockage in the flow of intestinal contents [2, 3]. It increases morbidity and mortality [4, 5]. The burden of intestinal obstruction in Ethiopia ranges from 21.8% to 4.6% [6, 7].

Despite the advancements in the field of medicine, introduction of a safe surgery checklist, improved monitoring and related safety practices during anesthesia, surgical technique, and conservative management, the surgical management outcome of intestinal obstruction remains a challenge to the healthcare system [1]. Surgical care is followed by fatal and nonfatal postoperative complications from the diseases itself, the operation, and the anesthesia [8]. Globally, the World Health Organization (WHO) 2019 fact sheet on healthcare-associated infections revealed that a hundred million patients were affected by healthcare-associated infections, each year. Point prevalence ranges from 3.5 to 12% in developed and 5.7 to 19.1% in low- and middle-income countries [9]. The burden of healthcare-associated infections was also reported in sub-Saharan Africa [10], Botswana (13.4%) [11], South Africa (8%) [12], and Ethiopia (13-35.8%) [13–16].

Universally, intestinal obstruction varies from country to country in terms of incidence and management outcomes depending on ethnicity, age group, dietary habits, residence, geographic location, the living condition of the community, presentation, length of hospital stay, comorbid illness, duration of operation, duration of illness, presence of peritonitis, and service provision [5, 17–19]. Difficulties in using the checklist, postoperative intra-abdominal infections, the inadequacy of training, and insufficient amount of anesthesiologists, nurses, and support staff [19–22] were some of the challenges which lead to poor management outcomes.

In this case, few studies were conducted in north and central Ethiopia referral hospitals related to the pattern of admissions [6, 23]; there is a paucity of research on predictors of surgical outcome of intestinal obstruction in Ethiopia, particularly in regional hospitals. Thus, this study was conducted to assess clinical outcomes and predictors of intestinal obstruction surgery in Chiro General Hospital, Eastern Ethiopia.

## 2. Methods and Materials

### 2.1. Study Design, Period, Setting, and Population

An institutional-based cross-sectional study was conducted in Chiro General Hospital, Eastern Ethiopia. Chiro Town is situated at 328 km to the east of Addis Ababa. The hospital provided healthcare service for more than 1,441,008 populations in its catchment area with a total of 166 beds. All patients surgically treated for intestinal obstruction from January 1, 2014, to December 31, 2017, were the study populations. A total of 254 patients suffering from intestinal obstruction were included in the study.

### 2.2. Data Collection Methods

Data were collected based on a structured data abstraction sheet from medical records and registers. The abstraction sheet includes sociodemographic factors, type of procedure, and duration of illness. The data was extracted from medical charts. The data was collected by 3 BSc nurses and 1 MSc clinical midwifery supervisor. Completeness of each recording format was checked before collecting the data.

### 2.3. Variables

#### 2.3.1. Dependent Variable

The surgical management outcome is considered the dependent variable (poor, good).

#### 2.3.2. Independent Variables

Sociodemographic characteristics (age, sex, and residence), duration of illness, cause of obstruction, procedure done, and intraoperative findings are the independent variables.

#### 2.3.3. Operational Definitions

Surgical treatment means surgical exploration of the abdomen which is determined by the nature of obstruction [1].

Poor management outcome is the condition of the patient after the procedure has been done where the patient develops postoperative complications (dehiscence, surgical site infection, pneumonia, and shock) or died until the patient is discharged from the hospital [2].

#### 2.3.4. Data Quality Control

The pretest was done on 5% of the sample size in Felege Hiwot Referral Hospital. One-day training was given for data collectors and supervisors on data collection tools and data collection procedures. Supervision and completeness of each abstraction sheet had been checked by the principal investigator and the supervisors on a daily basis. Checking for double data entry was done by two data clerks, and the consistency of the entered data was cross-checked.

#### 2.3.5. Data Processing and Analysis

Data were entered, coded, cleaned, and checked by EpiData statistical software version 4.2.0.0, and analysis was done using SPSS version 22 statistical software. Descriptive statistics was presented using tables, figures, and texts. Binary logistic regression was used for analysis. During bivariable analysis, seven variables with a *p* value < 0.25 were considered for multivariable logistic regression analysis. The odds ratio along with 95% CI was estimated to identify factors associated with the outcome variable. The level of significance was declared at a *p* value ≤ 0.05.

## 3. Results

### 3.1. Sociodemographic Characteristics of Study Participants

A total of two hundred fifty-four patients participated in the study. The mean age of the participants was 34 years (SD ±16.24). The majority (226, 89%) were males (Table 1).

### 3.2. Clinical Presentation, Duration, and Preoperative Diagnosis of Intestinal Obstruction

**Figure 1:**
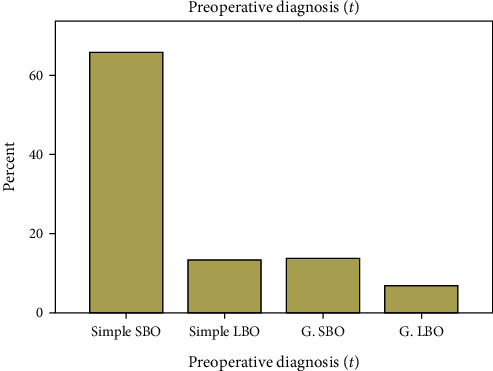
Clinical diagnosis of surgically treated patients with intestinal obstruction in Chiro General Hospital, 2018 (*N* = 254). SBO: small bowl obstruction; LBO: large bowl obstruction; G: gangrenous.

All patients presented with the clinical symptoms of abdominal pain, whereas 245 (96.6%), 242 (95.3%), and 232 (91.3%) patients present with vomiting, abdominal distension, and failure to pass flatus and feces, respectively. In addition, 8.7% of patients had a history of groin swelling. 65.7% were diagnosed with simple small bowel obstruction (SBO). On the other hand, 13.4% were diagnosed as having simple large bowel obstruction (LBO) (Figure 1).

### 3.3. Intraoperative Finding and Surgical Procedures Done

Almost half of admissions (47.6%) were due to small bowel volvulus, followed by 16.5% adhesion and bands and 13.8% sigmoid volvulus. Derotation and decompression (DD) and resection and anastomosis (RA) surgical procedures were done for 42.1% and 29.5% of patients, respectively (Table 2).

### 3.4. Magnitude of Poor Management Outcome

The magnitude of poor surgical management outcome of intestinal obstruction was 21.3% (95% CI: 16.5-26.4). More than half (55.5%) had wound site infection (hematoma and incisional surgical site infection), 14.8% postoperative pneumonia, and 11.1% anastomotic leak.

### 3.5. Factors Associated with Poor Management Outcome

A binary logistic regression was done to identify the association between the poor outcome of intestinal obstruction and independent variables. In the bivariable analysis, age ≥ 55 years, out of Chiro residence, duration of illness ≥ 24 hrs, preoperative diagnosis of gangrenous SBO and gangrenous LBO, the operative finding of gangrenous small bowel volvulus, and the operative procedure of DD and RA were identified. However, in multiple logistic regression analysis, the age group of ≥55 years, duration of illness of ≥24 hours, preoperative diagnosis of gangrenous SBO, and gangrenous LBO were significantly associated with poor surgical outcomes.

Patients with the age of ≥55 years were nearly 3 times more likely to develop poor outcomes as compared with patients whose age was ≤55 years (AOR = 2.9, 95% CI: 1.03, 8.4). Patients who came late (≥24 hours) were about three times more likely to develop poor outcomes compared with patients who came early (<24 hours) (AOR = 3.1, 95% CI: 1.03, 9.4). Those patients with gangrenous LBO and gangrenous SBO had, respectively, 3.6 and 4.2 times higher odds of developing unfavorable outcome than patients with simple SBO (AOR = 3.6, 95% CI: 1.3, 9.8 and AOR = 4.2, 95% CI: 1.3, 13.7, respectively) (Table 3).

## 4. Discussion

Intestinal obstruction is the surgical emergency followed by fatal and nonfatal postoperative complications. While surgical intervention is intended to save the lives of individuals, unsafe surgical care can cause substantial harm to the patient.

The magnitude of poor management outcomes of intestinal obstruction was 21.3%. This study is in line with the study done in Adama (24.6%) [6] and India (25.89%) [24]. But the magnitude in this study is lower than those in studies conducted in Canada (64%) [25] and Nigeria (66.5%) [26]. This might be due to differences in the cause, type of procedure done, and study population. Intussusception was the most common cause of intestinal obstruction in Nigeria. On the contrary, it was higher than the study done in Kenya (13.6%) [27]. The possible reason might be due to the difference in the place of residence. In Kenya, 58.7% of patients came from rural dwellers [27], whereas 78% in the current study. It is believed that patients who came from the urban area could have good awareness on the importance of getting health service earlier. The other possible reason might be the difference in the standard of surgical procedures.

In the current study, old age, late presentation of illness, and preoperative diagnosis of a gangrenous bowel were significantly associated with the occurrence of poor outcomes. Surgical site infection threatens the lives of millions of patients each year and contributes to the spread of antibiotic resistance bacteria (*Staphylococcus aureus*, *Escherichia coli*, and *Pseudomonas aeruginosa* [28, 29]. In the current study, wound site infection (hematoma and incisional SSI) was found to be the major poor surgical outcome (55.5%). In addition, postoperative pneumonia and anastomotic leak were reported among 14.8% and 11.1% patients, respectively. Similarly, SSI was reported in Adama, Kenya, Botswana, and Nigeria which accounts for 39.3%, 33%, 9%, and 31.4%, respectively [6, 26–28]. The incidence of SSI can be reduced by administering perioperative antibiotics such as ampicillin, cefotaxime, metronidazole, and amoxicillin/clavulanate. Literature showed that patients with an acute abdomen should receive preoperative antibiotics and postoperative antibiotics in case of perforation [30, 31]. One study showed no significant effect on the postoperative outcome by administering metronidazole for perforated appendicitis [32]. Perioperative antibiotic administrations depend on different factors such as anatomic region undergoing the specific surgical procedure, timing of surgery, age of the patient, time of antibiotic administration, urgency of the procedure, and availability of the drugs. Surgical antibiotic administration after incision was associated with a significantly higher incidence of SSI compared with administration before incision [33, 34]. So, the burden of SSI can be minimized by applying WHO recommendations [35].

Patients aged ≥55 years were more likely to develop poor management outcomes compared with those patients whose ages are less than 55 years. The study is in line with studies done in Gondar [17], Japan [36], and China [37]. This is true, as age increases the physiologic process of organs and tissue progressively degenerates over time [38] and decreased immune response [39].

Patients who came late were more likely to develop poor outcomes than patients who came earlier. This is consistent with studies conducted in Adama [6] and Gondar [17]. This might be due to poor health-seeking behavior and a poor transportation system in this subregion. Late presentation in the case of intestinal obstruction accounts for disastrous outcomes, notably a high rate of complications, long hospital stay, and high mortality rates [40]. Identifying which patient needs early surgery is difficult, given the lack of specific clinical or radiographic signs [41]. Moreover, clinical presentation of surgical problems in the elderly may be subtle, and handling stress poorly because of physiological change may lead to delay in diagnosis.

## 5. Conclusion

The magnitude of poor management outcomes was high. Old age, late presentation of illness, and gangrenous bowel obstructions were significantly associated with poor surgical outcome. Emphasis should be given in improving the patient's outcome using a surgical patient safety checklist and creating awareness in seeking care for emergency conditions and postoperative complications. In addition, effective infection prevention activities have to be implemented in the hospital setting. Future research should be done on barriers of delay to take care for surgical illnesses in a prospective manner by including variables such as educational status, occupational status, income, and knowledge-related factors.

## Figures and Tables

**Table 1:** Sociodemographic distribution of patients surgically treated for intestinal obstruction in Chiro General Hospital, 2018.

Category	Frequency	Percent
Age		
<55	222	87
≥55	32	13
Sex		
Male	226	89
Female	28	11
Residence		
Chiro	56	22
Out of Chiro	198	78

**Table 2:** Intraoperative finding and surgical procedures done for patients with IO who were treated surgically in Chiro General Hospital, 2018 (*N* = 254).

Variables	Frequency	Percent (%)
Intraoperative finding		
Small bowel volvulus	212	47.6
Adhesion and bands	42	16.5
Sigmoid volvulus	35	13.8
Intussusception	22	8.7
Strangulated hernia	21	8.3
Others	13	5.1
Type of procedures done		
Derotation and decompression	107	42.1
Resection and anastomosis	75	29.5
Adhesiolysis & band release	38	15.0
Herniorrhaphy	13	5.1
Hartmann's colostomy	10	3.9
Reduction	9	3.5
Other procedures	6	2.4

**Table 3:** Factors associated with poor surgical management outcomes of intestinal obstruction surgery in patients admitted to CGH, 2018.

Variables	Surgical outcome		COR: 95% CI	COR: 95% CI
	Poor (%)	Good (%)		
Age				
≥55	12 (37.5)	20 (62.5)	2.6 (1.2-5.7)	2.9 (1.03-8.4)^∗^
<55	42 (18.9)	180 (81.1)	1	1
Residence				
Out of Chiro	48 (24.2)	150 (75.8)	2.7 (1.1-6.6)	2.7 (0.9-7.6)
Chiro	6 (10.7)	50 (89.3)	1	1
Duration of illness				
≥24 hours	49 (28.3)	124 (71.7)	6 (2.3-15.7)	3.1 (1.03-9.4)^∗^
<24 hours	5 (6.2)	76 (93.8)	1	1
Preoperative diagnosis of IO				
Simple LBO	6 (17.6)	28 (82.4)	1.7 (0.6-4.6)	1.5 (0.5-4.5)
Gangrenous SBO	21 (60.0)	14 (40.0)	11.7 (5.1-26.7)	3.6 (1.3-9.8)^∗^
Gangrenous LBO	8 (44.4)	10 (55.6)	6.2 (2.2-17.7)	4.2 (1.3-13.7)^∗^
Simple SBO	19 (11.4)	148 (88.6)	1	1
Intraoperative procedure done				
DD	9 (8.4)	98 (91.6)	0.2 (0.1-0.5)	0.7 (0.3-1.9)
Other procedures	45 (30.6)	102 (69.4)	1	1
RA	34 (45.3)	41 (54.7)	6.6 (3.4-12.6)	2.0 (0.8-5.3)
Other procedures	20 (11.2)	159 (88.8)	1	1
Intraoperative finding				
Gangrenous SBV	19 (59.4)	13 (40.6)	7.8 (3.5-17.2)	2.1 (0.7-6.2)
Other findings	35 (15.8)	187 (84.2)	1	1

^∗^Significant at a *p* value < 0.05; 1 is the reference. SBO = small bowel obstruction; LBO = large bowel obstruction; DD = derotation and decompression; RA = resection and anastomosis; SBV = small bowel volvulus; SV = sigmoid volvulus.

## Data Availability

All relevant data are within the manuscript.

## Abbreviations

AOR: Adjusted odds ratio
CGH: Chiro General Hospital
COR: Crude odds ratio
CHMS: College of Health and Medical Sciences
GLBO: Gangrenous large bowel obstruction
GSBO: Gangrenous small bowel obstruction
HU: Haramaya University
IHRERC: Institutional Health Research Ethics Review Committee
IO: Intestinal obstruction
LBO: Large bowel obstruction
RA: Resection and anastomosis
SBO: Small bowel obstruction
SBV: Small bowel volvulus
SOP: Standard of procedure
SSI: Surgical site incision/infection.
